# Functional interaction between posterior cerebellar lobes and secondary somatosensory cortex during somatosensory mismatch detection

**DOI:** 10.1162/imag_a_00572

**Published:** 2025-06-04

**Authors:** Virginie Destrebecq, Antonin Rovai, Nicola Trotta, Xavier de Tiège, Gilles Naeije

**Affiliations:** Department of Neurology, CUB Hôpital Erasme, Hôpital Universitaire de Bruxelles (H.U.B.), Université Libre de Bruxelles (ULB), Brussels, Belgium; Laboratoire de Neuroanatomie et Neuroimagerie translationnelles (LN ^2^ T), UNI – ULB Neuroscience Institute, Université Libre de Bruxelles (ULB), Brussels, Belgium; Department of Translational Neuroimaging, CUB Hôpital Erasme, Hôpital Universitaire de Bruxelles (H.U.B.), Université Libre de Bruxelles (ULB), Brussels, Belgium

**Keywords:** somatosensory processing, predictive coding, cortico-cerebellar connectivity, cortico-cerebellar loops, cerebellar brain inhibition

## Abstract

According to the predictive coding theory, the brain predicts future sensory inputs based on previous experiences. When there is a mismatch between the expected and the actual stimulus, a mismatch response is transmitted from low to high cortical levels to adapt the predictive model. An important cortical area for somatosensory mismatch responses is the secondary somatosensory (S2) cortex, which has reciprocal connections with the cerebellum. This study aims to characterize the role of the cortico-cerebellar interactions in the modulation of S2 cortex responses according to the predictability of afferent tactile and proprioceptive somatosensory inputs. We enrolled 20 right-handed healthy adults who underwent three functional magnetic resonance imaging (fMRI) runs (6 min each, block-design) consisting of twelve 30-s alternating blocks (10 brain volumes/block, 120 brain volumes/session) of tactile oddball paradigms. The fMRI signals within the contralateral S1 (cS1), ipsilateral S2 (iS2), and contralateral S2 (cS2) cortices; within the cortical areas involved in multimodal sensory mismatch detection (i.e., the right anterior insula (AIns), temporoparietal junction (TPJ), middle frontal gyrus (MFG), and supplementary motor are/anterior cingular cortex (SMA/ACC)); and within the ipsilateral cerebellar lobule 8 (iCL8) and 6 (iCL6) were extracted using region-of-interest (ROI) analyses and compared using ANOVA. The modulation of cortico-cerebellar functional connectivity by afferent stimuli predictability was studied using psychophysiological interaction (PPI) analyses. Predictable tactile stimuli were associated with significantly lower fMRI signals within cS1 and bilateral S2 cortices, and the right AIns, TPJ, and MFG compared to random tactile stimuli. PPI analyses showed that predictable tactile stimuli were associated with a significant increase in the functional connectivity (negative correlation) between cS2 cortex and iCL8 BOLD levels, with no significant correlation during random tactile stimulation. This effect, identified by the PPI analyses, occurred solely between the cerebellum and cS2 cortex and not with the other cortical mismatch areas. This study provides evidence for a cerebello-cortical interplay between iCL8 and the cS2 cortex in tactile somatosensory mismatch responses. The lower BOLD response in the S2 cortex observed for predictable tactile stimuli is likely mediated by an inhibitory influence of the cerebellum on the somatosensory cortex when tactile inputs are predictable.

## 1 Introduction

Sensory systems are continuously exposed to a multitude of sensory inputs. In order to operate safely and efficiently within the environment, salient sensory stimuli must be swiftly identified to elicit appropriate behavioral responses. According to the predictive coding theory (for reviews, seeFicco et al., 2021;Friston, 2010;Friston & Kiebel, 2009;Teufel & Fletcher, 2020), this process is subserved by the ability of the human brain to generate and update predictions about upcoming sensory inputs based on previous experiences. Under this framework, top-down predictions are made by high-level associative neocortical areas and confronted at lower cortical levels with sensory inputs. When there is a mismatch between the expected and the actual stimulus, a prediction error is detected, and a mismatch neural response is transmitted from lower to higher cortical levels to adapt the predictive model.

Previous studies have identified that cortical sensory mismatch responses involve modality-specific cortical areas hierarchically subordinated to a network of multimodal cortical areas activated across sensory inputs (Allen et al., 2016;Downar et al., 2000;Grundei et al., 2023a). For the somatosensory modality, specific mismatch responses mainly involve the secondary somatosensory (S2) cortex and less consistently the primary somatosensory (S1) cortex contralateral (cS1) to the stimulation (Allen et al., 2016;Downar et al., 2000;English et al., 2023;Fardo et al., 2017;Gijsen et al., 2021;Grundei et al., 2023a;Huang et al., 2005;Naeije et al., 2016;Ostwald et al., 2012). Cortical multimodal mismatch response areas typically involve the anterior insula (AIns) (Allen et al., 2016), the temporo-parietal junction (TPJ) (Downar et al., 2000;Grundei et al., 2023a), the middle frontal gyrus (MFG), and the supplementary motor area/anterior cingulate gyrus (SMA/ACC), preferentially in the right hemisphere (Allen et al., 2016;Downar et al., 2000;Fardo et al., 2017;Grundei et al., 2023a;Naeije et al., 2016).

In addition to this neocortical network, there is mounting neuroimaging evidence suggesting that the cerebellum, and more precisely the cerebellar posterior lobes (Bushara, 2001), is also involved in somatosensory mismatch responses. Tactile stimuli lead to increased activity in the cerebellar posterior lobes in functional magnetic resonance imaging (fMRI) (Bushara et al., 2001) and their blood oxygen level dependent (BOLD) responses are lower when tactile stimulations are self-generated, and thus more predictable, compared to externally produced stimuli (Blakemore et al., 1999;Kilteni & Ehrsson, 2020). Second, the BOLD signal in the cerebellar dentate nuclei, the main efferent tract of the cerebellum, is increased during passive cutaneous stimulation, but it doubles when the same cutaneous stimulation is associated with a change detection task (Gao et al., 1996). Third, the cerebellar posterior lobes display higher BOLD levels when the expected somatosensory outcome fails to occur in hand-reaching tasks (Schlerf et al., 2012). This finding is paralleled by magnetoencephalography (MEG) studies, which have shown that the cerebellum displays evoked responses to tactile omissions, in a manner similar to the S2 cortex (Andersen & Dalal, 2021;Tesche & Kahru, 2000). Evidence also suggests that cortical somatosensory mismatch responses could involve a cortico-cerebellar interplay. Neuroanatomically, animal studies have identified projections from parietal associative cortices to the cerebellar cortex in cats (Oka et al., 1979;Sasaki et al., 1975) and monkeys (Clower et al., 2001;Kelly & Strick, 2003;Schmahmann & Pandya, 1989). Human resting-state fMRI studies have paralleled animal data and highlighted reciprocal connections between the posterior cerebellum and the parietal lobes (Buckner et al., 2011;Habas, 2021;O’Reilly et al., 2010;Sang et al., 2012;Stoodley & Schmahmann, 2010;Strick et al., 2009). Functionally, transcranial direct current stimulation of the cerebellar cortex (ctDCS) modulates the amplitude of mismatch responses in the S2 cortex elicited by spatial discrimination and vibratory somatosensory oddballs (Chen et al., 2014). Finally, patients with cerebellar lesions display altered S2 cortex mismatch responses to less predictable tactile stimuli compared with healthy subjects (Naeije et al., 2019;Restuccia et al., 2007).

Still, based on the aforementioned studies, it is difficult to capture the exact functional role of the interactions between the cerebellum and cortical structures for somatosensory mismatch responses. Our hypothesis is that the cerebellum acts on the somatosensory cortical mismatch network by a mechanism akin to the “cerebellar brain inhibition” framework (CBI) described for the primary motor (M1) cortex (Galea et al., 2009;Grimaldi et al., 2016). The CBI theory postulates that the cerebellum exerts a predominantly inhibitory drive on cortical areas. Specifically, cerebellar efferent connections to the M1 cortex consist in a tri-synaptic pathway that starts with Purkinje Cells (PC). PCs inhibit large excitatory interneurons in the dentate nuclei (DN), which, in turn, target excitatory interneurons in thalamic nuclei that connect to neocortical areas. Increased PC activity induces cortical inhibition by reducing the dentato-thalamic facilitatory drive to cortical areas. Upon activation of cerebellar PC by ctDCS, motor thresholds required to generate motor-evoked potentials (MEPs) increase and MEPs amplitude are reduced (Benussi et al., 2021;Daskalakis et al., 2004). This phenomenon was coined CBI and indicates that activation of Purkinje cells results in inhibition of the M1 cortex (Galea et al., 2009;Oldrati & Schutter, 2018;Ugawa et al., 1991). We posit that, within a similar framework, the cerebellum might inhibit the cortical somatosensory mismatch detection network, and especially the S2 cortex, when confronted with predictable tactile stimuli.

The aim of this fMRI study is thus to better characterize the interplay between the cerebellum and the cortical network involved in somatosensory mismatch responses. Based on previous functional neuroimaging results, we expected that the activity of the cortical structures involved in somatosensory mismatch detection would be significantly increased in response to less predictable somatosensory stimuli compared to more predictable ones. We also hypothesized that these effects would be associated with changes in the functional connectivity between the cerebellar posterior lobes and the cortical areas modulated by the predictability of afferent tactile stimuli.

## 2 Materials and Methods

### 2.1 Participants

Twenty healthy adults (9 males, 11 females, mean age: 35.5 years, range age: 26–61 years, SD: 9.81) without neurological comorbidities and antiepileptic/psychotropic drug consumption were enrolled in this study after signing written informed consent. All subjects were right-handed (mean score 0.7, range score 0.4–1) according to the Edinburgh handedness Inventory (Oldfield, 1971).

This study was carried out at the CUB Hôpital Erasme (Brussels, Belgium) after approval by the institutional Ethics Committee (P2019/491-CCB: B406201941751).

### 2.2 Experimental paradigms

#### 2.2.1 Oddball paradigm

The oddball paradigms are illustrated inFigure 1.

**Fig. 1.**
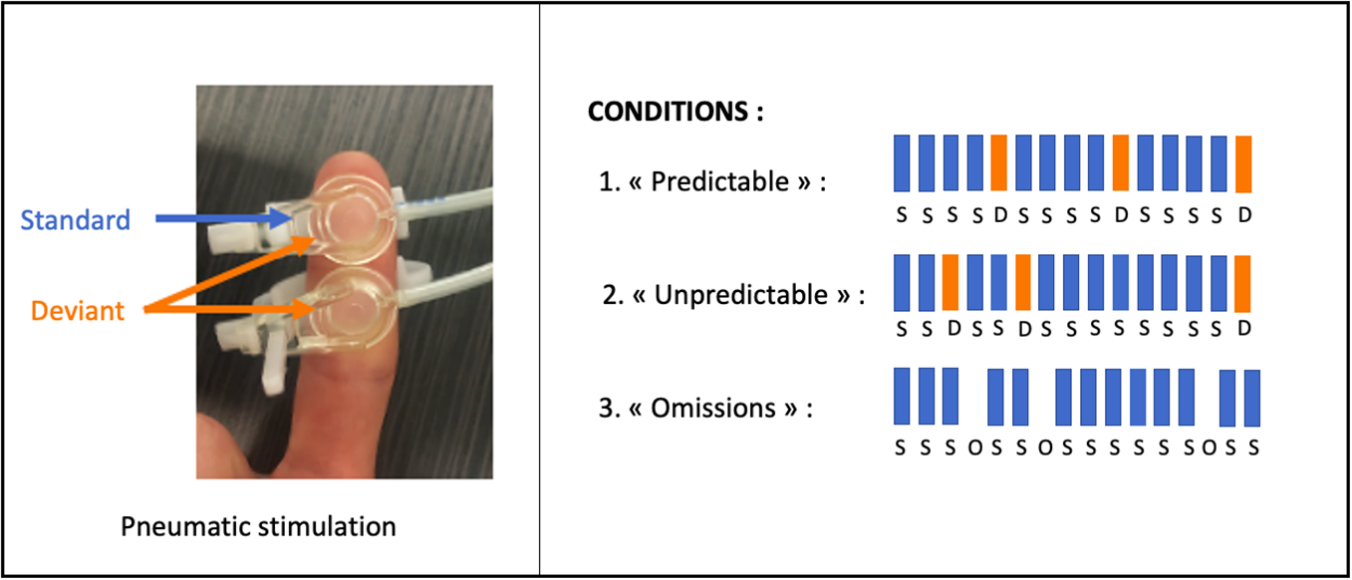
Illustration of the Oddball paradigms used in this study employing pneumatic tactile stimulation. Standard stimuli were applied to the right index fingertip, while deviant stimuli consisted of double stimulations applied simultaneously to the right index fingertip and the middle phalanx.

To explore the effect of the predictability of afferent somatosensory stimuli on the cerebellar and cortical networks involved in somatosensory mismatch responses, we used three oddball tactile paradigms derived from (Naeije et al., 2017,^2023^), hereafter referred to as “*oddball conditions*”. Tactile stimuli were applied using an MRI-compatible pneumatic stimulator (Wienbruch et al., 2006). Standard stimuli (*Standards*) were applied to the right index fingertip (stimulation area: 1 cm², intensity: 3.5 bars, duration: 100 ms, interstimulus interval: 500 ms), while deviant stimuli consisted either of double stimulations applied simultaneously to the right index fingertip and the middle phalanx (*Deviants*) or consisted in stimulus omission (*Omissions*).

Pneumatic tactile stimuli were chosen over peripheral nerve electrical stimulation because they are more natural, avoid activating a broad range of fibers with varying conduction velocities, and bypass peripheral mechanoreceptors and distal nerve segments (Naeije et al., 2019). Additionally, electrical stimuli can be perceived as painful, potentially engaging the cortical pain matrix, which overlaps with sensory mismatch detection areas (Allen et al., 2016;Naeije et al., 2017). Standards and deviants were applied to the same finger in order to recruit as many common peripheral and cortical pathways as possible (Naeije et al., 2019). Of note, although deviants stimulate more mechanoreceptors than standards, we showed, in a previous study using the same stimulation protocol, that change detection responses in the somatosensory cortices were similar when the roles of standards and deviants were reversed (Naeije et al., 2016).

The predictability of deviant occurrence was manipulated according to the following conditions: in the “*Predictable*” condition,*Deviants*always occurred after four*Standards*; in the “*Unpredictable*” condition,*Deviants*were randomly interspersed between*Standards*; and in the “*Omission*” condition, the deviance consisted of*Omissions*. The*Standards/Deviants*ratio was set at 0.8 in the*Predictable*and*Unpredictable*conditions (Naeije et al., 2017), and the*Standards/Omissions*ratio was set at 0.95 corresponding to the ratio used in a previous auditory and somatosensory oddball study that used a similar paradigm (Naeije et al., 2016,^2017^,^2023^;Wacongne et al., 2011;Wager et al., 2013). If cerebellar and cortical areas involved in change detection responses are modulated by the predictability of afferent tactile inputs, we expected similar increases in BOLD signal in the*Unpredictable*and*Omission*conditions compared to the*Predictable*condition.

### 2.3 fMRI data acquisition

All MRI data acquisitions were performed on a hybrid 3 T SIGNA PET-MRI scanner (GE Healthcare, Milwaukee, Wisconsin, United States) using a 24-channel head and neck coil. Functional data were acquired using single-shot gradient-echo echo-planar imaging (GE/EPI) T2* weighted images, covering the whole brain (repetition time [TR]/echo time [TE]/flip angle [α], 3.000 msec/34 msec/90°; field of view, 28 cm; acquired matrix, 96 × 96; slice thickness, 3 mm; in-plane resolution, 2.9 × 2.9 mm; 40 interleaved slices). Four dummy scans (total duration, 12 s) were obtained before each session to allow the MR signal to reach a steady state and were subsequently automatically discarded by the scanner. A three-dimensional T1-weighted BRAVO sequence covering the whole brain was acquired in all subjects to co-register the functional data (TR/TE/α: 8.3 msec /3.1 msec/12°; field of view: 24 cm; matrix: 240 × 240; slice thickness: 1 mm; in-plane resolution 1 × 1 mm), as in (Naeije et al., 2023). All subjects underwent 3 functional runs (1 run per condition). Each run was designed as a 6 min block-design fMRI paradigm consisting of twelve 30 s alternating blocks of different task/rest conditions (10 brain volumes per block, 120 brain volumes per paradigm) as in (Destrebecq et al., 2023). The order of the conditions was fully randomized across participants, and the total procedure time was 18 min.

### 2.4 Data analysis

fMRI data preprocessing and analyses were performed using the SPM12 software (Wellcome Department of Imaging Neuroscience, London, UK;https://www.fil.ion.ucl.ac.uk/spm/), implemented in Matlab (2017a; MathWorks Inc., Sherborn, MA, USA) using a conventional pipeline detailed in (Destrebecq et al., 2023). Functional images were preprocessed using a conventional SPM approach, which included realignment, unwarping, slice-timing correction, co-registration to the subjects’ corresponding structural images, normalization into the Montreal Neurological Institute (MNI) space, and smoothing—obtained by applying an isotropic Gaussian kernel of 8 mm full-width at half maximum (FWHM). A high-pass filter was applied to remove signal drifts with a period longer than 128 s.

#### 2.4.1 First level analysis

After preprocessing, statistical analyses were performed in the general linear model (GLM) framework. For each subject, we constructed one GLM for each condition that included the preprocessed fMRI data in which the experimental conditions (e.g., oddball conditions vs. rest) were modeled as boxcar functions convolved with the canonical hemodynamic response function. The GLMs also included, as co-variates of no interest, the corresponding six motion parameters obtained from realignment. First-level (within-participant) contrasts for each condition against the baseline (rest period) were generated to search for significant increases in BOLD signals elicited by the experimental conditions.

#### 2.4.2 Second-level analysis

These analyses aimed to identify the group-level increases in BOLD signal induced by oddball conditions compared to rest in order to ensure that they, indeed, recruited the ROIs described in the literature for somatosensory cortical mismatch response and for cerebellar areas associated with hand tactile stimulation. For that purpose, the individual contrast images, normalized in MNI space, issued from the first-level analyses corresponding to the three oddball conditions were entered into a GLM for the second-level analyses that was based on a random effects model. A One-sample*t*-test was then used to assess group-level increases in BOLD signal in the oddball conditions. The significance threshold for the resulting statistical map was set at p < 0.05 corrected for the family-wise error (FWE) rate (extent threshold k = 10) (Allen et al., 2016;Downar et al., 2000;Grundei et al., 2023a).

#### 2.4.3 ROI analysis

We used an ROI approach to investigate how the level of the BOLD signal in cerebellar and cortical areas previously demonstrated to be involved in somatosensory mismatch responses is affected by the predictability of somatosensory afferences. For that purpose, we identified, from the literature, ROIs in cortical areas that have consistently been described across fMRI sensory oddball studies studying sensory mismatch responses (Allen et al., 2016;Destrebecq et al., 2023;Downar et al., 2000;Grundei et al., 2023a) and selected the ROIs involved in both somatosensory change detection and in mismatch responses across multiple sensory modalities (Allen et al., 2016;Chen et al., 2014;Downar et al., 2000;Grundei et al., 2023a;Naeije et al., 2017,^2023^;Restuccia et al., 2007). We included in these ROI analyses the somatosensory cortical areas (cS1 cortex and S2 cortices contra- (cS2) and ipsilateral (iS2) to the tactile stimulation) as identified by previous finger tactile stimulation studies (Destrebecq et al., 2023;Eickhoff et al., 2006) as well as the following multimodal change detection areas: AIns, MFG, SMA/ACC, and TPJ of the right hemisphere (Allen et al., 2016). Finally, we included cerebellar lobule 6 (iCL6) and lobule 8 (iCL8) ipsilateral to the tactile stimulations in our ROI analyses. This decision was based on previous reports indicating that lobule 6 and lobule 8 are involved in finger somatosensory attenuation (Kilteni & Ehrsson, 2020), somatosensory touch prediction (Andersen & Lundqvist, 2019), and sensory prediction errors (Schlerf et al., 2012). Additionally, both lobules contribute to hand sensorimotor representation (Bushara et al., 2001;Nettekoven et al., 2024) and are functionally connected to S2 cortex in resting-state fMRI studies (Buckner et al., 2011;Bushara et al., 2001;Nettekoven et al., 2024). ROIs were built using the MarsBaR toolbox for SPM12 and defined as 5 mm radius spheres built around MNI coordinates reported in the literature. These MNI coordinates are: [−58, −20, 46] for cS1 cortex based onDestrebecq et al., 2023, [-49, -19, 18] for cS2 cortex, and [51, -21, 19] for IS2 cortex based on Eickhoff et al., 2006. For the multimodal areas involved in change detection, ROIs were centered on the MNI coordinates identified byAllen et al. (2016), in their effective connectivity model of hierarchical processing of tactile mismatch response, which described a right-dominant network that included: the right MFG (rMFG) [36, 50, 22], the right SMA/ACC (rSMA/ACC) [3, 23, 43], the right AIns (rAIns) [36, 20, 1], and the right TPJ (rTPJ) [54, -40, 22] paralleling previous fMRI (Downar et al., 2000) and MEG (Naeije et al., 2016) findings. Finally, for the cerebellar ROIs, we selected MNI coordinates located within the hand representation areas of iCL6 [24, -51, -23] and iCL8 [16, -70, -56] from Nettenkoven’s hierarchical atlas of the human cerebellum (Nettekoven et al., 2024). These coordinates are also included in the iCL6 and iCL8 regions implicated in somatosensory error prediction paradigms (Schlerf et al., 2012), in the finger sequence task of the Multi-Domain Task Battery Functional Atlas (King et al., 2019), as well as in the iCL6 and iCL8 areas exhibiting resting-state functional connectivity with the parietal operculum (Buckner et al., 2011). This selection was made to best reflect the specific characteristics of our paradigm.

Comparisons of the mean activities in each cerebellar and cortical ROIs between the three different oddball conditions were performed using one-way ANOVAs using JASP (JASP Team, 2019). When a significant (p < 0.05) main effect was reached, we applied a Bonferroni correction (p-values were adjusted for N = 9 ROIs) (Han & Glenn, 2018). When the result remained significant, post hoc*t-*tests were conducted.

#### 2.4.4 Psychophysiological interaction analyses

*Psychophysiological interaction (*PPI) analyses indicate if the contribution of a given brain area to the level of activity in another brain area changes significantly with an experimental factor (Friston et al., 1997;Grundei et al., 2023a). Based on our working hypothesis, we used PPI analyses to search for an effect of somatosensory expectations on the contribution of the BOLD signal at cerebellar ROIs to those of the rest of the brain. We expected significant changes in functional connectivity between the cerebellum and the cortical somatosensory expectation violation network, and especially with the cS2 cortex. In practice, PPI analyses were performed for each cerebellar ROI (iCL6 and iCL8) using a seed-to-whole-brain approach (for details on the theoretical framework and methods underlying PPI analyses; seeFriston et al., 1997;Toiviainen et al., 2020;Zhang et al., 2022). The two seeds used for PPI analyses were exactly the same spheres used for the ROI analysis. For each participant and each seed ROI, time series were extracted as physiological variables and entered into a first-level analysis based on a PPI-specific GLM with the condition onset times (one PPI-GLM for each condition) as psychological regressors, in order to contrast each oddball condition (A total of 6 contrasts were explored: Predictable > Unpredictable, Unpredictable > Predictable, Omission > Predictable, Predictable > Omission, Unpredictable > Omission, and Omission > Unpredictable). The individual contrast images were then entered into a second-level analysis, in which task-dependent PPI effects were investigated at the group level using a one-sample*t*-test. We reported significant clusters using a threshold of p < 0.05 after correction for multiple comparisons at the whole-brain level or using a small volume correction (SVC) p < 0.05 with a 10 mm radius spherical volume of interest centered on our predetermined ROIs if they emerged from the seed-to-whole-brain analyses. Finally, when a significant PPI was found, we illustrated the observed PPI effects between the cerebellar ROIs and the corresponding cortical area(s) by generating a regression plot (done with Matlab) of their mean BOLD signals according to the experimental conditions (Friston et al., 1997;Trotta et al., 2016) in which the difference in regression slope represents the significant interaction effect.

## 3 Results

### 3.1 Group-level analyses

The tactile oddball conditions led to the expected significant increases in BOLD signal in a neural network that included the cS1 cortex, the iS2 and cS2 cortices, the rIFG, the rMFG, the rSMA/ACC, as well as the iCL8.

### 3.2 ROI analysis

ROIs are illustrated inSupplementary Material Figure S1in which ROIs are superimposed on the group-level activation map.

In the oddball conditions, one-way ANOVA applied to the level of BOLD signal in cS1 and bilateral S2 cortices revealed significant differences between the oddball conditions (F = 5.618, p = 0.046, iS2 cortex; F = 5.918, p = 0.032, cS2 cortex, F = 5.527, p = 0.042, cS1 cortex). Post-hoc*t*-Test found significantly higher increases in BOLD signal in bilateral S2 cortices and cS1 cortex in the*Unpredictable*condition compared with the*Predictable*condition (cS2 cortex, 0.89±0.15 vs. 0.60±0.11, p = 0.004,*t*= -3.347; iS2 cortex, 0.78±0.16 vs. 0.45±0.11, p = 0.003,*t*= -3.459; cS1 cortex 0.84 ±0.12 vs. 0.56±0.13, p = 0.003,*t*= 5.445). There was no significant difference between the*Unpredictable*and the*Omission*conditions at S2 and S1 cortices (cS2 cortex, 0.89±0.15 vs. 0.72±0.16, p = 0.213,*t*= -1.500, BF10 = 0.78, iS2 cortex, 0.78±0.16 vs. 0.62±0.16, p = 0.281,*t*= -1.511, Bayesian Factor (BF) 10 = 1.02, cS1 cortex, 0.84±0.12 vs. 0.62±0.11, p = 0.118,*t*= -1.304, BF10 = 0.95) nor between the*Omission*and the*Predictable conditions*(cS2 cortex, 0.72±0.16 vs. 0.60±0.11, p = 0.407,*t*= 1.264, BF10 = 0.84; iS2 cortex, 0.62±0.16 vs. 0.45±0.11, p = 0.251, t = -1.295, BF10 = 0.89, cS1 cortex, 0.62±0.11 vs. 0.56±0.13, p = 0.408,*t*= 1.045, BF10 = 1.10). Results are illustrated inFigure 2A.

**Fig. 2.**
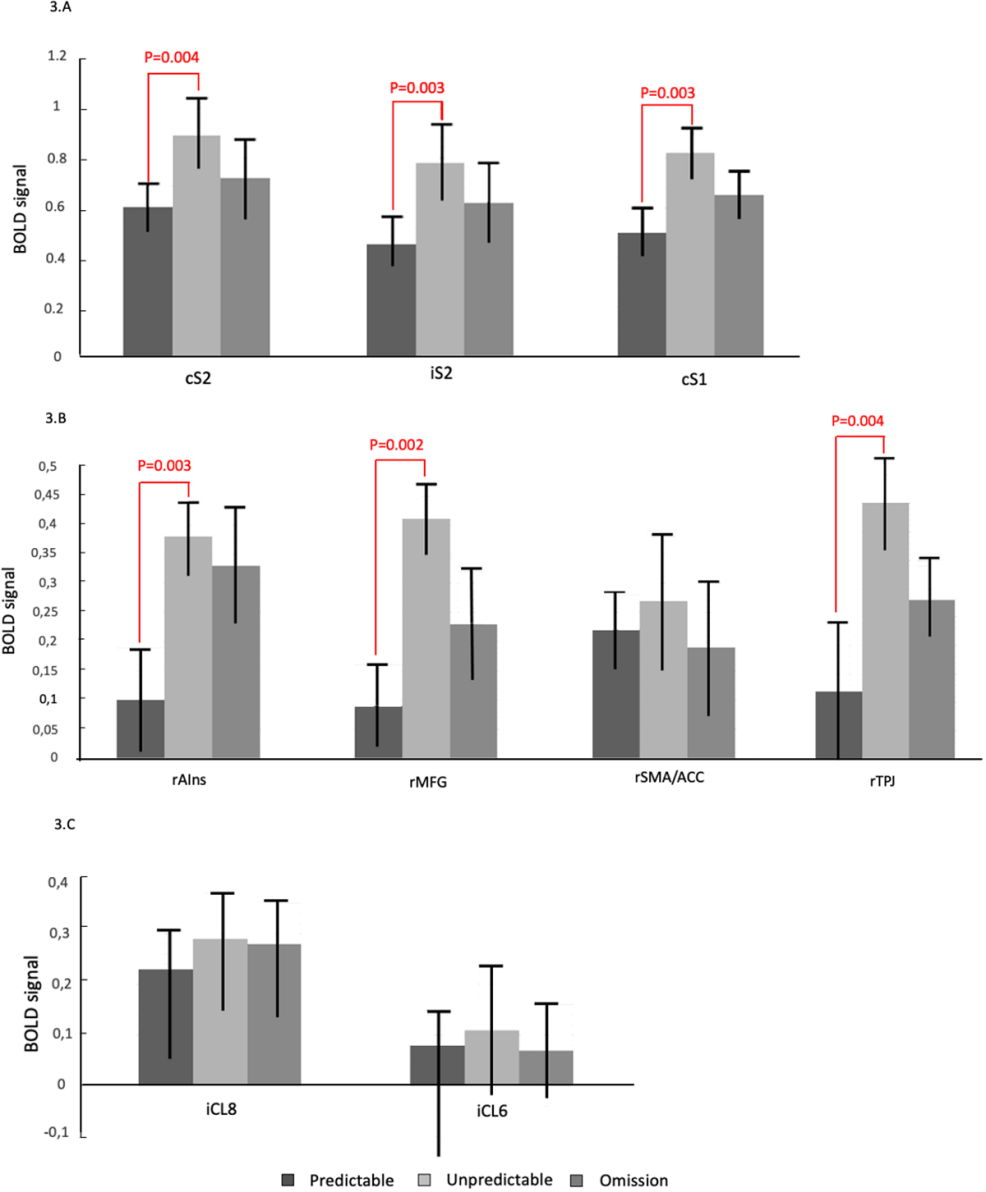
(A, B, C) Bar plots of the contrast estimates per condition in cS2, iS2, cS2, rSMA/ACC, rAIns, rMFG, iCL6, and iCL8. Errors bars indicate 90% CIs. Activities in bilateral S2, cS1, rAIns, and rMFG were significantly higher in the “Unpredictable” condition of the oddball paradigm. No significant differences were found between the other oddball conditions. No significant differences were found within the cerebellar ROIs between the different oddball conditions.

One-way ANOVA applied to the BOLD signal of the other cortical areas involved in somatosensory change detection revealed significant differences between the oddball conditions for the rAIns (F = 5.456, p = 0.042), the rMFG (F = 6.044, p = 0.028), and the rTPJ (F = 4.926, p = 0.037). Post-hoc t-test found significantly higher increases in BOLD signal in rAins, rMFG, and the rTPJ in the*Unpredictable*condition compared with the*Predictable*condition (rAIns, 0.38±0.07 vs. 0.10±0.09, p = 0.003,*t*= -2.510; rMFG, 0.41±0.06 vs. 0.09±0.07, p = 0.002,*t*= 3.630; rTPJ, 0.45±0.07 vs. 0.13±0.11, p = 0.004,*t*= 5.967) and there was no significant difference between the*Unpredictable*and the*Omission*conditions (rAIns, 0.38±0.07 vs. 0.33±0.1, p = 0.702,*t*= 1.800, BF10 = 0.97; rMFG, 0.41±0.06 vs. 0.23±0.09, p = 0.124,*t*= 7.700, BF10 = 1.12; rTPJ, 0.45±0.07 vs. 0.27±0.1, p = 0.113,*t=6.902*, BF10 = 0.82) nor between the*Omission*and the*Predictable*conditions (rAIns, 0.33±0.1 vs. 0.10±0.09, p = 0.154,*t*=*-*0.723, BF10 = 1.22; rMFG, 0.23±0.09 vs. 0.09±0.07, p = 0.505,*t*= 1.292, BF10 = 0.93, rTPJ, 0.27±0.1 vs. 0.13±0.11, p = 0.753,*t*= 2.504, BF10 = 0.96) for all of these cortical ROIs. Finally, there was no statistical difference in BOLD signal within the rSMA/ACC according to the Oddball condition (F = 0.223, p = 0.423, BF10 = 1.07). Results are illustrated inFigure 2B.

For the iCL8 and iCL6, there was no statistical difference in BOLD signal between the oddball conditions (F = 0.419, p = 0.360, BF10 = 0.94, iCL8, F = 0.397, p = 0.39, BF10 = 1.06, iCL6). Results are illustrated inFigure 2C.

### 3.3 PPI analyses

Based on the above results, we performed the PPI analysis to search for a significant difference in the contribution of the BOLD signal at the iCL8 ROI to the rest of the brain between*Predictable*and Unpredictable oddball conditions. The results of this analysis are available in the Supplementary Materials (Table S1). Using small-volume correction, we found a significant PPI effect in the contralateral S2 cortex as the only area involved in somatosensory mismatch detection. When tactile stimuli were more predictable, there was a significant change in the contribution of iCL8 to the level of the BOLD signal in cS2 cortex compared to when stimuli were less predictable. These results are illustrated inFigure 3and4for the cS2 cortex and the iCL8, andFigure 4Cshows the changes in interaction (regression slopes) between the activity measured in the cS2 cortex and iCL8 according to the predictability of the tactile deviants (*Predictable*vs*. Unpredictable*). Correlation analyses disclosed a significant negative correlation between the level of the BOLD signal in iCL8 and cS2 cortex in the Predictable condition (r = -0.8126, p = 0.0016), meaning that there is an inverse relationship between their BOLD signals while there was no significant correlation between cS2 cortex and iCL8 in the Unpredictable condition (r = -0.2518, p = 0.1089). No significant change (even at p < 0.001 uncorrected) in functional connectivity between the cS2 cortex and iCL8 was observed for the*Omission*condition compared with the other oddball conditions (*Predictable*vs*. Omission, Unpredictable*vs*. Omission*). No significant PPI effect with the other cortical areas involved in somatosensory mismatch detection was found.

**Fig. 3.**
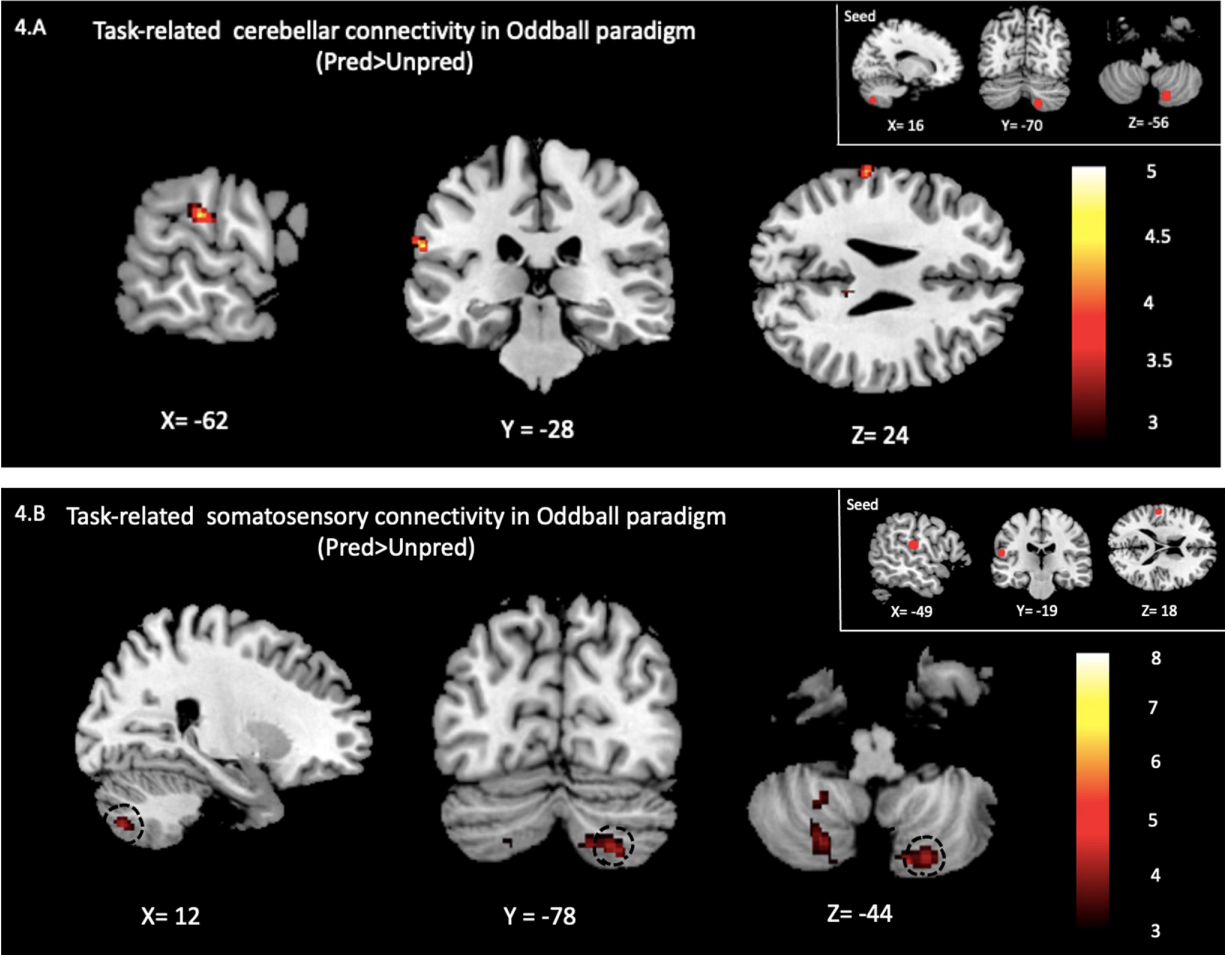
Cortical and cerebellar peaks showing increased connectivity with the seeds of interest in the “Predictable” compared to the “Unpredictable” condition of the oddball paradigm. (A) Sagittal (left), coronal (middle), and axial (right) views of the significant peak (p < 0.05 FWE-corrected) in the cS2 that increased its connectivity with iCL8 (seed). (B) Sagittal (left), coronal (middle), and axial (right) views of the peaks in the cerebellum (left crus1, left lobule 8, and right lobule 8) that increased their connectivity with the cS2 (seed). The significant (p < 0.05 FWE-SVC-corrected) activity is black-circled (right lobule 8: iCL8). For visualization and illustration purposes, all activation maps were thresholded at p < 0.001 uncorrected for and overlaid on ch2bet template provided by MRIcron.

**Fig. 4.**
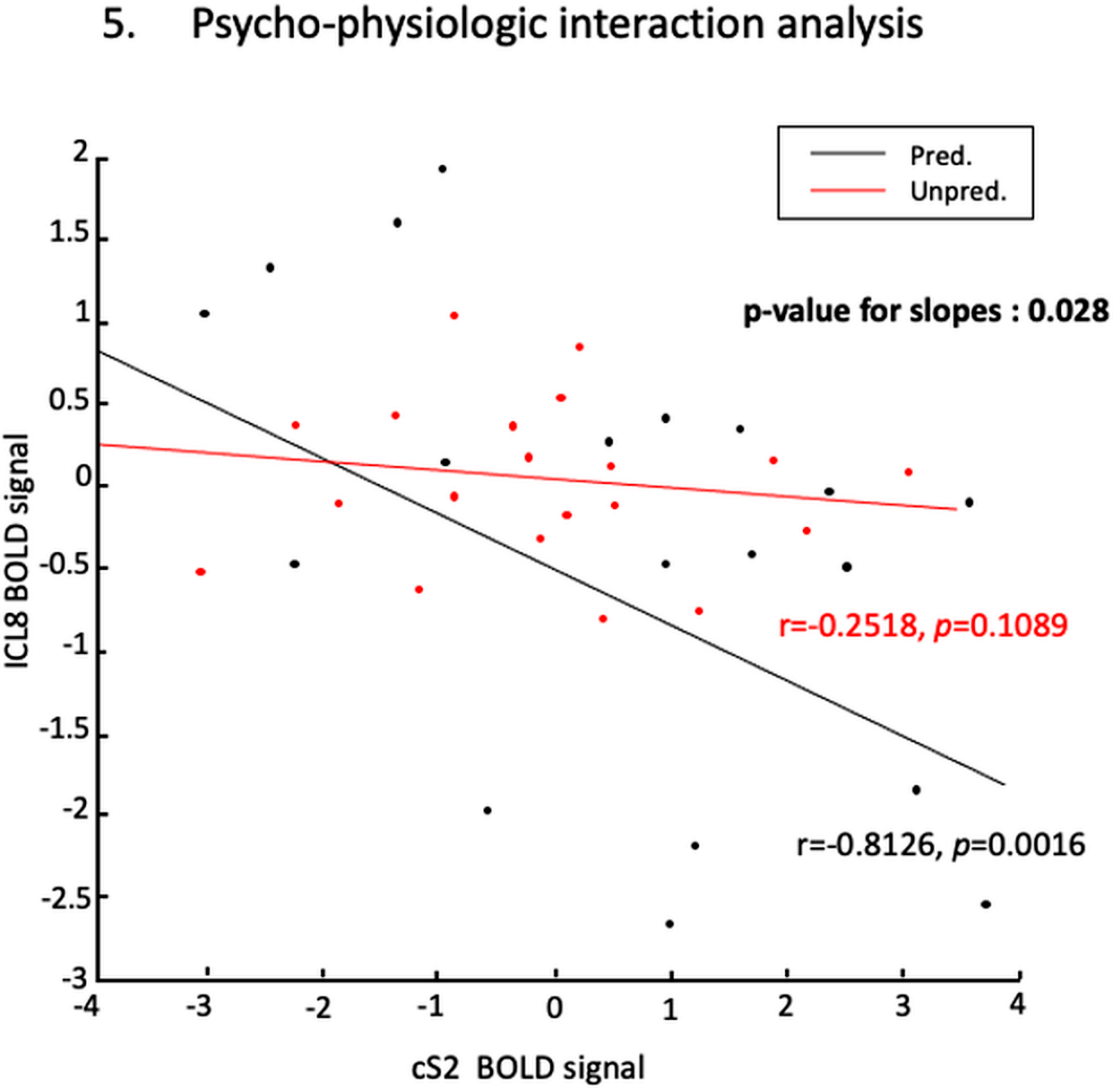
Regression plots illustrating the mean activity (mean BOLD signal) in ICL8 and the cS2 in the “Predictable” condition (black plots) and the “Unpredictable” condition (red plots). Note the difference between the regression slopes relative to both experimental conditions as assessed by the SPM psychophysiological interaction analysis reflecting the increase of functional connectivity between both brain areas in the specific context of a predictable stimulation.

For iCL6, no significant PPI effect (even at p < 0.001 uncorrected) was found with cortical areas involved in somatosensory mismatch detection when contrasting the different oddball conditions. This analysis was also performed for the local maximum within the cerebellar lobule 6 [32, -62, -26] to exclude spurious negative findings from ROI selection, and did not yield significant results. These results are available in the Supplementary Materials (Table S2 and S3).

## 4 Discussion

This study shows that the modulation of the predictability of tactile stimuli affects the activity of nodes of the cortical somatosensory mismatch detection network that includes the somatosensory cortices as well as multimodal cortical areas such as the rAIns, rMFG, and rTPJ (i.e., the lower the predictability, the higher the activity). The predictability of tactile stimuli specifically modifies the functional connectivity between the cerebellar posterior lobe (iCL8) and the S2 cortex, with a significant negative correlation when deviants are highly predictable. This could indicate that the cerebellum modulates activity in the cS2 cortex when tactile inputs are more predictable.

### 4.1 Somatosensory mismatch detection network

In the oddball conditions, the recruited neural network for somatosensory mismatch detection was in line with previous fMRI, EEG, and MEG tactile and multimodal sensory oddball studies that described a cortical network involved in sensory mismatch detection across sensory modalities, which includes SMA, IFG, MFG, AIns, and TPJ, preferentially in the right hemisphere (Allen et al., 2016;Downar et al., 2000;Grundei et al., 2023a;Pessoa & Ungerleider, 2004;Rusiniak et al., 2013;Stevens et al., 2005;Strobel et al., 2008). Both the S1 and S2 cortices also displayed increased BOLD signals for less predictable tactile stimuli. This finding was expected as S2 cortex is known to process elaborate somatosensory stimulus features such as timing, spatial location, pain, and their surprising aspect (Chen et al., 2008,^2010^;Fujiwara et al., 2002;Hämäläinen et al., 2002;Ostwald et al., 2012;Simões et al., 2002;Zhu et al., 2007) and because somatosensory mismatch responses at S2 cortex are consistently found in somatosensory oddball tasks across functional neuroimaging techniques (fMRI, MEG, EEG) (Downar et al., 2000;English et al., 2023;Fardo et al., 2017;Gijsen et al., 2021;Grundei et al., 2023a,2023b;Naeije et al., 2016;Ostwald et al., 2012). Mismatch responses in the S1 cortex are also regularly found in fMRI (Allen et al., 2016;Grundei et al., 2023a), MEG (Manganotti et al., 2009;Xu et al., 2021), and EEG studies (Grundei et al., 2023b) when deviant stimuli are associated with a different (Akatsuka et al., 2007) intensity than the standard stimuli, but not when the mismatch concerns the stimulus location or texture (Downar et al., 2000). This is supported by fMRI studies that showed that variation in an afferent stimulus intensity directly affects the evoked responses in the S1 cortex more than in the S2 cortex (Akatsuka et al., 2007;Backes et al., 2000; Fereti et al., 2007;Hlushchuk et al., 2015;Mauguière et al., 1997) and suggests that mismatch responses in the S1 cortex are more likely to reflect intensity variation than the processing of prediction errors*per se*.

In our study, the activity of the posterior cerebellum was not directly modulated by the predictability of the tactile stimulus. Nevertheless, the increase in BOLD signal observed at iCL8 during our tactile oddball conditions corresponds to its involvement in sensorimotor processing, as described in fMRI studies (Backes et al., 2000;Buckner et al., 2011;Hari et al., 1993;Hlushchuk et al., 2015;Mauguière et al., 1997;Nelson et al., 2004;Stoodley & Schmahmann, 2010;Wager et al., 2013; Wikstom et al., 1996). Moreover, an fMRI study investigating multimodal sensory expectation violation identified an increase in posterior cerebellar activity elicited by somatosensory oddball stimuli (Grundei et al., 2023a). The modification of the mutual functional connectivity between iCL8 and S2 cortex for predictable stimuli supports a role for CL8 in tactile prediction. This finding parallels previous MEG investigations (Andersen & Lundqvist, 2019) that found activity in iCL8 using an oddball paradigm where deviant stimuli consisted of omissions. The involvement of iCL8 found in this study is also in line with a seminal fMRI tactile change detection study that showed higher activity in the cerebellar dentate nuclei, suggesting an increased recruitment of the cerebellar cortex upstream (Gao et al., 1996). Similarly, fMRI studies on error prediction associated with a motor task, which have used a change detection paradigm in the visual modality (Pessoa & Ungerleider, 2004) or an auditory oddball (Rusiniak et al., 2013;Stevens et al., 2005;Strobel et al., 2008), also reported the task-related recruitment of the posterior cerebellum, including iCL8. Overall, these findings concur to support a role for the posterior cerebellum in sensory change detection.

### 4.2 Effects of predictability on S2 cortex and cerebellar activities in oddball conditions

The level of the BOLD signal was lower in somatosensory cortices in the*Predictable*condition than in the*Unpredictable*condition, during which the occurrence of tactile deviants was less predictable. Still, only the cS2 cortex showed significant changes in functional connectivity with iCL8 according to tactile stimulation predictability. The role of S2 cortex in somatosensory mismatch responses is in line with previous EEG and MEG studies that investigated the neural responses elicited by tactile deviants as a function of their predictability, and which disclosed higher neural response amplitudes in the S2 cortex for less predictable somatosensory stimuli (Downar et al., 2000;Grundei et al., 2023a;Zhang et al., 2022). Our results contrast, however, with previous fMRI studies that failed to disclose any significant modulation of S2 cortex activity by deviants in an electrical oddball paradigm, where the intensity of the median nerve electrical stimulation differed between standard and deviant stimuli (Chen et al., 2008), or by vibratory tactile stimuli with predictable versus unpredictable intensity (Nelson et al., 2004). This can be explained by the fact that the first study used large (2 s) interstimulation intervals (ISIs) between electrical stimuli. A 2 s ISI corresponds to rare stimuli that escape most predictability attenuation in the S2 cortex (Hari et al., 1993;Wikstrom et al., 1996), likely blunting the detection of an additional modulation of its activity by deviants (Akatsuka et al., 2007,^2008^; Ferreti et al., 2007). In the other fMRI studies, the prediction variability only concerned the intensity of the stimulation, and intensity discrimination tasks do not usually enhance the S2 cortex BOLD signal in fMRI somatosensory paradigms (Allen et al., 2016;Grundei et al., 2023b). Here, the*Omission*condition did not lead to any significant modulation of S2 cortex BOLD signal compared to the*Predictable*condition, in accordance with previous fMRI and MEG studies that showed that the absence of an expected stimulation led to S2 cortex responses similar to the ones observed for deviants (Grundei et al., 2023b;Naeije et al., 2023;Ostwald et al., 2012). Omission responses have been described both by contrasting rare omissions with expected somatosensory stimulation (Andersen & Lundqvist, 2019), as in our study, and by contrasting rare omission with expected omissions in auditory paradigms (Wacongne et al., 2011). Such a contrast between predictable and unpredictable omission has, to our knowledge, never been studied in the somatosensory modality but it would allow to refine the understanding of the S2 cortex response to omissions and maybe highlight an implication of the cerebellum that was missed in our study due to lack of a predictable omission condition. Altogether, our results further support a role of the S2 cortex in neural responses, reflecting its role in somatosensory prediction.

Our study did not disclose the hypothesized modulation of the iCL8 according to the predictability of tactile stimuli. This may partly be explained by the relatively small sample of subjects, which may have reduced the sensitivity to detecting subtle variations in cerebellar BOLD signal levels according to stimulus predictability or significant BOLD signal changes for omitted stimuli. Alternatively, the use of a block-design paradigm that is less sensitive than event-related paradigms for identifying stimulus-specific BOLD signal modulations might also have played a role (Kannurpatti et al., 2011). However, an fMRI tactile event-related paradigm would have implied ISIs larger than 2 s, which would have prevented the study of tactile predictions in the tactile modality. Yet, importantly, functional connectivity analyses showed that when tactile stimuli were predictable, there was a significant contribution of iCL8 BOLD levels to cS2 cortex BOLD levels with a negative correlation between cS2 cortex and iCL8 BOLD levels. This result supports a role for iCL8 in the modulation of cS2 cortex activity, where the lower cS2 cortex activity observed when tactile stimulations are more predictable could be driven by an increased inhibitory drive from iCL8. These functional connectivity findings mirror those obtained byKilteni and Ehrsson (2020), who found increased cS2 cortex-cerebellar connectivity associated with attenuation of touch perception during self-generated somatosensory stimuli; a situation where proprioceptive somatosensory inputs are more predictable, compared with externally generated stimulation. This relation fits with the presumed function of the cerebellum in generating representations of incoming sensory stimulations, as well as with cerebellar neuroanatomy. The cerebellum is considered to be involved in forward computation models that are used to regulate cortical networks and associated behaviors (Leggio & Molinari, 2015). These computational predictive models are dynamically updated thanks to the confrontation of top-down and bottom-up sensory information in the cerebellar cortex (Leggio & Molinari, 2015;Naeije et al., 2019). The anatomical basis of this regulation lies in the reciprocal connection of neocortex to assemblies of cerebellar PC via the cortico-ponto-cerebellar and the dentato-thalamo-cortical pathways (Bushara et al., 2001;Guell et al., 2018;Nettekoven et al., 2024;Stoodley & Schmahmann, 2010;Stoodley et al., 2012). We argue that predictable somatosensory stimulation is detected and processed in the cerebellar cortex and leads to an increase of the CBI toward the S2 cortex, reducing its activity level. This interpretation is also supported by an fMRI study that observed reduced S2 cortex activity related to tactile oddball stimulation after an anodal (i.e., excitatory) cerebellar transcranial direct current stimulation (ctDCS), which activates PC (Forss & Jousmäki, 1998). Electrophysiological studies have also shown that the difference in the amplitude of somatosensory evoked responses for unpredictable compared with predictable stimuli was reduced by a cerebellar lesion (Restuccia et al., 2007) or cerebellar inhibition by cathodal ctDCS or continuous theta burst stimulation (cTBS) (Wasaka et al., 2007), while excitatory anodal ctDCS had the opposite effect (Chen et al., 2014). Interestingly, the cerebellum cortical topographic organization is characterized by multiple representation maps in distinct lobules for sensorimotor and cognitive processes. Task and resting-state fMRI investigations brought evidence for a double representation of body maps for tactile and motor stimuli and a triple representation for cognitive tasks such as language, working memory, and emotion processes (Buckner et al., 2011;Guell et al., 2018). Tactile stimuli are associated with two distinct bodymaps, one in the cerebellar lobule 5 (CL5) and the other in CL8 (Bushara et al., 2001;Wiestler et al., 2011). The biological significance of these multiple representation remains mostly unknown but clinical evidence suggests that their role may not be equal as acute lesions within CL5 or CL8 lead to different impairments (Stoodley et al., 2016). Our data, along with those ofKilteni and Ehrsson (2020)that showed that the cerebellum posterior lobe representation is involved in tactile attenuation, suggest that the CL8 tactile representation could be involved in afferent tactile stimuli prediction processes.

A limitation of our paradigm could be the short ISI (500 ms) chosen between tactile stimuli, which prevented an event-related analysis centered around deviant as chosen in previous fMRI tactile oddball studies that used an ISI of 2 s for standards and between 6–34 s for deviants (Chen et al., 2008;Hari et al., 1993). The Short ISI and block design could possibly account for the absence of significant changes in the BOLD signal of CL8 in relation to stimulus predictability or omissions. However, ISIs that allow the study of time-locked responses in fMRI are typically in the second range and exceed the ISI that would permit the study of attenuation or mismatch responses at the S2 cortex (Mauguière et al., 1997;Naeije et al., 2017). The lack of behavioral tasks limits our ability to determine if the interaction between iCL8 and cS2 cortex is linked to tactile perception attenuation. Further study using electrophysiological methods that allow to study stimulus evoked responses with ISI < 500 ms and sufficient spatial discrimination like cryogenic (Andersen et al., 2020) or optically-pumped magnetoencephalography (Lin et al., 2019) combined with a behavioral task could help to better characterize the interplay between the cerebellum and S2 cortex in somatosensory processing and perception.

In conclusion, this study provides evidence for a cerebello-cortical interplay between CL8 and the S2 cortex in tactile somatosensory mismatch responses. Specifically, CL8 contributes to the attenuation of S2 BOLD levels for predictable tactile stimuli through increased functional connectivity when tactile stimuli are predictable. In somatosensory mismatch responses, the CL8 seems to have a main inhibitory effect on the S2 cortex, which is consistent with the CBI framework.

## Supplementary Material

Supplementary Material

## Data Availability

The raw data supporting the conclusions of this article will be made available by the authors, after approval by institutional authorities (Université libre de Bruxelles & Hôpital Universitaire de Bruxelles).
