# Supplementary material for: Functional interaction between posterior cerebellar lobes and secondary somatosensory cortex during somatosensory mismatch detection
Source: Imaging Neurosci (Camb). 2025 Jun 4;3:imag_a_00572. doi: 10.1162/imag_a_00572 (PMC12319935; doi:10.1162/imag_a_00572)
Supplement: Supplementary Material [file imag_a_00572-supp.pdf]

<u>Figure 2</u>

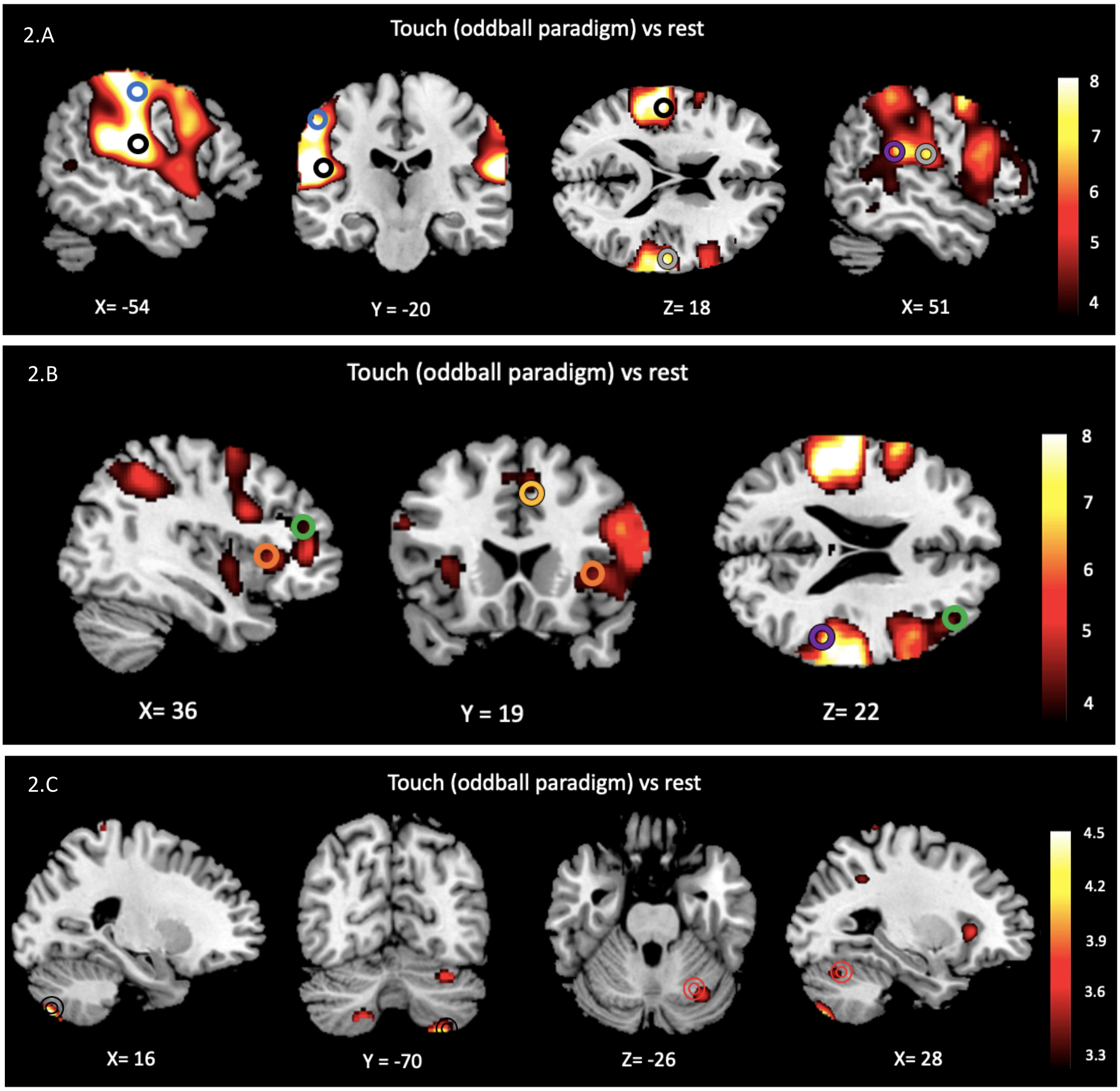

Significant increases in BOLD signal at somatosensory, multimodal cortical and cerebellar areas during oddball paradigms (all conditions grouped together) compared with rest. ***2.A,*** *Slice views of cS2 (black-circled), iS2 (grey-circled), cS1 (blue-circled) and TPJ (purple-circled) ROIs.* ***2.B,*** *Slice views of and rSMA/ACC (yellow-circled), rAIns (orange-circled, rMFG (green-circled) and TPJ (purple-circled) ROIs.* ***2.C****, Slice views of significant activities (p<0,05FWE) within the cerebellum: significant cerebellar clusters were found within the ipsilateral lobule 8, ipsilateral crus1 and contralateral lobule 8. Cerebellar ROIS are illustrated: iCL8 (black-circled) and iCL6 (red-circled). For visualization and illustration purposes, all activations maps were thresholded at p<0.001 uncorrected and overlaid on ch2bet template provided by MRIcron.*

Table 1. Activation peaks showing increased of connectivity with the cerebellar area (iCL8 ROI) according to the condition of the oddball paradigm.

Condition	Anatomical Region	MNI coordinates (mm)			T-value	Z-score	p-value
		x	y	z			
Predictible >Random	L S2 (supramarginal gyrus)	-62	-26	24	4.27	3.31	0.031 FWE-corrected *
	R post. cingulum	10	-42	30	3.93	3.13	0.001 uncorrected
Random >Pedictible	R angular gyrus	34	-64	48	4.14	3.25	0.001 uncorrected
	R temporal sup gyrus	66	-42	18	4.06	3.20	0.001 uncorrected
Predictible>Omission	L caudate nuclei	-16	-16	22	3.83	3.11	0.001 uncorrected
Omission> Random	R Paracentral lobule	10	-22	68	3.94	3.21	0.003 uncorrected
Omission>Predictible	No supratreshold cluster (p<0.01 unc)						
Random > Omission	No supratreshold cluster (p<0.01 unc)						

For each peak activation, the coordinates in MNI space, the t-value, z-value and the p-value are reported. We denote that a peak survived a threshold of p < 0.05 after correction for multiple comparisons at the whole brain or small volume (*) by the term "FWE-corrected" following the p value. Alternatively, the term "uncorrected" follows the p value in the few cases when the activation did not survive correction for multiple comparisons, but it is still informative to describe.

Table 2. Activation peaks showing increased of connectivity with the cerebellar area (iCL6 ROI) according to the condition of the oddball paradigm.

Condition	Anatomical Region	MNI coordinates (mm)			T-value	Z-score	p-value
		x	y	z			
Predictible >Random	L mid Cingulum	-4	12	36	4.97	3.81	0.001 uncorrected
	R Sup frontal gyrus	26	-8	70	4.28	3.44	0.001 uncorrected
Random >Pedictible	L cerebellum (VI)	-4	-66	-20	4.17	3.38	0.001 uncorrected
	R temporal sup gyrus	66	-42	18	4.06	3.20	0.001 uncorrected
Predictible>Omission	No supratreshold cluster (p<0.01 unc)						
Omission> Random	No supratreshold cluster (p<0.01 unc)						
Omission>Predictible	No supratreshold cluster (p<0.01 unc)						
Random > Omission	No supratreshold cluster (p<0.01 unc)						

For each peak activation, the coordinates in MNI space, the t-value, z-value and the p-value are reported. We denote that a peak survived a threshold of p < 0.05 after correction for multiple comparisons at the whole brain or small volume (*) by the term "FWE-corrected" following the p value. Alternatively, the term "uncorrected" follows the p value in the few cases when the activation did not survive correction for multiple comparisons, but it is still informative to describe.

Table 3. Activation peaks showing increased of connectivity with the cerebellar area (iCL6 cluster maxima) according to the condition of the oddball paradigm.

Condition	Anatomical Region	MNI coordinates (mm)			T-value	Z-score	p-value
		x	y	z			
Predictible >Random	L mid Cingulum	-4	12	36	4.99	3.90	0.001 uncorrected
	R Sup frontal gyrus	26	-8	70	4.40	3.62	0.001 uncorrected
Random >Pedictible	L cerebellum (VI)	-4	-66	-20	4.24	3.51	0.001 uncorrected
Predictible>Omission	No supratreshold cluster (p<0.01 unc)						
Omission> Random	No supratreshold cluster (p<0.01 unc)						
Omission>Predictible	R Orbitofrontal cortice	24	38	-8	4.0	3.41	0.001 uncorrected
Random > Omission	No supratreshold cluster (p<0.01 unc)						

For each peak activation, the coordinates in MNI space, the t-value, z-value and the p-value are reported. We denote that a peak survived a threshold of p < 0.05 after correction for multiple comparisons at the whole brain or small volume (*) by the term "FWE-corrected" following the p value. Alternatively, the term "uncorrected" follows the p value in the few cases when the activation did not survive correction for multiple comparisons, but it is still informative to describe.
